# The civic integrationist turn in Danish and Swedish school politics

**DOI:** 10.1186/s40878-017-0049-z

**Published:** 2017-03-01

**Authors:** Christian Fernández, Kristian Kriegbaum Jensen

**Affiliations:** 1 Department of Global Political Studies, Malmö University, Malmö, Sweden; 2 Department of Political Science, Aarhus University, Aarhus, Denmark

**Keywords:** Civic integrationist turn, Citizenship education, Denmark, Sweden, Public philosophy, Mother tongue instruction, Civics, History, Religion

## Abstract

The civic integrationist turn usually refers to the stricter requirements for residence and citizenship that many states have implemented since the late 1990’s. But what of other policy spheres that are essential for the formation of citizens? Is there a civic turn in school policy? And does it follow the pattern of residence and citizenship? This article addresses these questions through a comparative study of the EU’s allegedly strictest and most liberal immigration regimes, Denmark and Sweden, respectively. The analysis shows a growing concern with citizenship education in both countries, yet with different styles and content. Citizenship education in Denmark concentrates on reproducing a historically derived core of cultural values and knowledge to which minorities are expected to assimilate, while the Swedish model subscribes to a pluralist view that stresses mutual adaptation and intercultural tolerance. Despite claims to the contrary, the analysis shows that Sweden too has experienced a civic turn.

## Introduction

Scholars studying the West European civic integrationist turn only rarely extend their analyses to include school policies. Instead, they have mainly focused on the increasing use of formalized language, knowledge, and employment requirements for permanent residence and citizenship since the late 1990s (Goodman, 2014; Joppke, 2007). Yet, increasing cultural diversity in schools has also created a push, especially from the EU, to reconsider whether current practices of citizenship education produce good liberal-democratic citizens (Faas, Hajisoteriou, & Angelides, 2014). This increased focus on bridging ethno-cultural diversity and conveying societal values and knowledge to coming generations of citizens could be viewed as part of a broader civic integrationist turn. West European politics has generally demonstrated an intensified concern with producing and re-producing a national citizenry with the skills and virtues required to sustain a well-functioning liberal democracy and social welfare state (cf. Mouritsen, 2008). This may involve a wide range of policies, from strict and carefully crafted entry requirements for newcomers to softer forms of value and knowledge transmittance to pupils in schools.

Citizenship education is usually distinguished from the vocational and professional ends of education. Simply put, the objective of the latter is to prepare children for a life as able and self-sustaining employees on the labor market, while the objective of the former is to *integrate* them into the national community by making them responsible, engaged, and politically literate participants in society. To this end, a strong sense of national belonging and understanding of the national culture is often perceived as crucial. All key issues when the national community discusses whether and how to change in response to increasing cultural diversity.

Yet, citizenship education is a quite different *kind* of integration policy area than permanent residence and naturalization. First, it does not single out immigrants but is focused on creating good citizens out of all children. Second, it is not tied to the achievement of rights and thus less likely to come into conflict with notions of social justice and fairness. Third, it cannot also be used as a tool for migration control and, finally, it involves children who unlike adult immigrants are not potential welfare recipients. All in all, it is an area of integration policy that is more purely about the national community and democracy, and less mixed up with economics, political and social rights, and migration. Thus, we cannot assume that policy-makers approach the two integration policy areas similarly.

Focusing on the recent two decades (early 1990s to the present), this article analyzes policy development in Danish and Swedish citizenship education in the primary and lower secondary levels of education with two aims. First, to investigate the ideological space within which Danish and Swedish policies on citizenship education have developed. What are the image(s) of the good, competent citizen—beyond working and paying taxes—that mainstream policy-makers want schools to pursue and by what means? To this end we analyze policy developments quite extensively and descriptively over time within each case and compare differences between the two cases. Second, in the concluding discussion, we compare the findings of the analysis to existing studies of Danish and Swedish politics of permanent residence and naturalization – the latter summarized below – and discuss the implications for how the civic integrationist turn and national models are theorized. The so called refugee crisis of the Fall 2015 and its potential effects on citizenship education in Sweden is *not* included in the analysis for the simple reason that it is too early to say if and how it will affect such education – apart from the obvious challenge of accommodating minors with no knowledge of Swedish.

The two cases under investigation, Denmark and Sweden, are both comprehensive welfare states in Northern Europe with similar political systems, experiences of post-War immigration, and democratic traditions. Yet their models or philosophies of *adult* immigrant integration differ substantially. Denmark has since the turn of the century implemented some of the most demanding requirements for residence and citizenship in Europe, while Sweden has not adopted any (Goodman, 2014). Somewhat simplified, the Danish approach can be described as restrictive and monocultural, in as much as minorities are expected to adapt and assimilate to mainstream culture in order to be fully incorporated and participating members of society. Citizenship is viewed as a reward for successful integration, the terms and content of which are decided by the state. While it is disputable whether the Danish conception of the nation is predominantly ethnic or civic (cf. Mouritsen & Olsen, 2013), it relies on a monist idea(l) of society in which Danish norms and values constitute the central reference point by which a political community is preserved and reproduced (Hedetoft, 2010; Jensen, 2014; Mouritsen, 2006). The Swedish model, by comparison (and equally simplified), is more liberal and multicultural, in as much as integration is viewed as an open-ended process of mutual adaptation between majority and minorities without predefined conditions and ends. Integration is not a condition for citizenship, but rather a voluntary process which is encouraged through rights, provisions and other forms of formal inclusion. While cultivating national identity is seen as important, there is no official set of Swedish norms and values, because integration is believed to equally rely on the majority’s ability to accept and adapt to new cultures (Borevi, 2014; Jensen, 2016; Soininen, 1999; Wiesbrock, 2011).^1^

The question pursued in the following pages, then, is whether the mono- and multicultural philosophies of integration outlined above are equally visible in Danish and Swedish citizenship education politics. The article is divided in three main parts. First, the analytical framework based on the concept of public philosophy of integration is presented along with its operationalization, and the data used to trace it. Second, we devote the greater part of the article to analyze political developments since the early 1990s within the teaching of mother language, religion, history and civics in compulsory education. Thirdly, we discuss the findings of the analysis, compare them to existing research on Danish and Swedish permanent residence and naturalization policies and discuss the implications for the theorizing of the civic integrationist turn and national models. We argue that two very different philosophies of (civic) integration dominate Danish and Swedish integration politics, irrespective of whether the issue is the schooling of children or the integration of adult immigrants.

## A public philosophy perspective

Adrian Favell (2001) argues that immigrant integration politics is generally structured by a deep-seated notion of social integration as something that is ‘encompassed, bounded and achieved by the historical nation state’ (2006, p. 52). Favell labels this particular perspective on national, social unity or cohesion an “amateur” public theory or philosophy of integration and defines its ideational structure as a combination of two parts: ‘a kind of functionalist social theory of what it is that holds nations together, with a normative political philosophy that expresses nationhood in terms of abstract civic values (usually citizenship)’ (2006, p. 51). That is, a public philosophy of integration consists of both a normative framework describing the conception of national identity that should be promoted in state policies *and* a causal framework describing what empirical processes the collective and individuals must undergo in order for the national community to retain or develop its (perceived) distinctiveness and cohesion. In the context of citizenship education politics, this can be reformulated as two essential questions: How is the future society, for which citizens-to-be are supposed to be prepared, imagined? And, how can that society be achieved through the schooling of young citizens?

A public philosophy presupposes a relatively stable ‘set of consensual ideas and linguistic terms held across party political lines’ (Favell, 2001, p. 2). Accordingly, in order to argue that different public philosophies have structured Danish and Swedish politics of citizenship education since the early 1990s, one has to show ideational stability behind the dominant policy patterns. Without stability in the sense that policy-makers do not simply change ideas in response to changing political and societal circumstances, there is only a weak basis for arguing that certain ideas are strongly embedded in the world-view of policy-makers. In addition to stability, one has to show that a significant share of policy-makers subscribe to the same ideas. Without agreement, there is no shared ideational basis on which policy discussions proceed.

Following Favell’s definition of a public philosophy of integration above, this paper distinguishes between two ideational levels on which to trace stability and consensus: normative and causal ideas. Assuming ideational stability, we propose distinguishing between four different strengths of a public philosophy (i.e., how politically dominating certain ideas are) depending on the level of political consensus concerning normative and causal ideas (see Table 1 below).^2^ The typology is original but meant as a heuristic device. Hence, it treats the dimensions as dichotomous even though they are in reality continuous. It addresses a need within the field to be more specific and systematic regarding what data must show in order to argue that a public philosophy is actually dominating national integration politics (Bertossi & Duyvendak, 2012).

**Table 1:** Determining the strength of a public philosophy

		Normative vision	
		Low, stable agreement	High, stable agreement
Causal ideas	Low, stable agreement	No public philosophy	Moderate public philosophy
	High, stable agreement	Weak public philosophy	Strong public philosophy

A public philosophy is *strong* over a given stretch of time if there is generally stable, high political agreement on both normative and causal ideas. Yet, the strength of a public philosophy is more dependent on agreement on the purpose of citizenship education policy (normative vision) than agreement on how to get there (causal ideas), because the relevance of causal ideas for policy-making depends on whether they can serve the preferred normative vision of society. Consequently, a *moderate* public philosophy is present when there is consensus on the normative vision but not on which public policies would serve that vision. A *weak* public philosophy is a situation in which there is consensus on the effects of different public policies, but little agreement on the normative vision to pursue and, hence, which public policies are most relevant. If there is little agreement on both the normative vision and causal ideas, there is no dominating public philosophy structuring politics (at that moment in time). This could, for example, be a situation where different political blocs compete to make their specific philosophy the dominating one.

Favell, along with other scholars categorized within the ‘national models’ tradition, have been criticized for treating national approaches to integration as dense, coherent, and stable, and thereby having a tendency to treat integration politics as rather depoliticized and depolarized (Bertossi & Duyvendak, 2012). The approach taken here does not assume such stability and coherence nor does it rule it out. Indeed, it might shift over time. Moreover, the presence of a strong public philosophy does not necessarily remove political disagreements. Showing that there is a strong ideational plateau determining the *kind* of social phenomena publicly problematized and the *kind* of policy solutions discussed still leaves open questions of degree as well as strategic considerations. For example, policy solutions may come in different versions or coalitional politics may necessitate a compromise. In other words, claiming that a certain public philosophy of integration is dominating is more a claim that political disagreements are relatively stable and coherent (over a given stretch of time) than there is political consensus on the law proposals presented in parliament.

In the analysis that follows, we are particularly interested in the normative and causal ideas used by Danish and Swedish political parties. How stable, distinct, and consensual are the answers offered to questions of citizenship education? The question is addressed in two steps: in the empirical analysis by offering a predominantly descriptive account of policy discussions and developments in the two countries; and in the conclusion by linking these developments to the typology above. Before turning to the analysis, however, a brief presentation of our data and method.

## Method and data

In the analysis, we trace the normative and causal arguments presented by political parties for different policy solutions. We record political agreement as high on a given issue when the government and major opposition party explicitly support the same ideas or, more implicitly, do not dispute the ideas presented by the other as the premises of policy discussions. We record stability when the normative and causal ideas evoked do not change considerably from one policy discussion to the next. In the analysis we trace how the policies and dominant ideas have developed since the late 1990s. We do not report the standpoints of all political parties throughout. Instead, we mainly report when there are disagreements.

The analysis focuses on significant national policy changes and debates regarding the subjects mother-tongue instruction, religion, history and civics/social science since the early 1990s. Citizenship education is approached holistically in both Danish and Swedish schools, i.e. as a democratic element that should be integrated in many subjects and school life in general rather than concentrated to one subject. Still, in practice it is mostly addressed through three subjects: religion, history and civics/social science. Because of their societal orientation, these subjects are generally viewed as particularly favorable and important for citizen-rearing activities. However, the analysis also includes mother-tongue and bilingual instruction. Although these are not citizenship-preparing activities per se, since they only apply to a minority of the students, they are closely intertwined with public conceptions of societal cohesion and (multi)cultural recognition, and therefore relevant to the wider theme of civic integration. In fact, the extent to which a political system supports linguistic diversity in schools offers an indication of its overall philosophy on integration (cf. Borevi, 2002, p. 182).

The analysis both draws on existing studies and committee reports, ministerial publications, parliamentary debates, and op-eds by Ministers and leading party members discussing the abovementioned subjects. All the material relating to major discussions since the early 1990s until 2015 has been collected and analyzed. Our selection of material is informed by previous research on education policy in combination with close survey of policy reforms and debates with due consideration taken to difference in policy process and style in the two countries. The use of committees to formulate policy problems and solutions are more common in Sweden and typically include representatives from the parliamentary parties. The few relevant Danish committees have only consisted of experts, and they have been tasked with designing solutions based on a government definition of the policy problem. Consequently, the Danish analysis is more oriented towards parliamentary debates and op-eds.

## Foundations of citizenship education in Denmark and Sweden

Today, the Danish and Swedish school systems are quite alike.^3^ They combine state centralization in the choice and prioritization of subjects and in the formulation of binding learning goals for the individual subjects with a high degree of teacher/school autonomy in deciding how to achieve those goals through teaching style and curriculum selection (Telhaug, Mediås, & Aasen, 2004). Since 1993, the Danish Minister of Education has the authority to determine obligatory topics to be covered, although it has only rarely been enforced.

Historically speaking, both countries share a commitment to comprehensive public schooling. The notion of a public, egalitarian school encompassing all strata of society is a fundamental tool to reduce the effect of socioeconomic inequalities on citizen’s education, professional careers, and political participation. The classic social democratic comprehensive school model, developed in tandem with other welfare institutions in the ‘golden decades’ of social reformism (roughly late 1940s to the early 1970s), puts emphasis on creating equal opportunities, a sense of national and social unity, and the democratic skills and knowledge required to effectively participate in democratic decision-making (Telhaug, Mediås, & Aasen, 2006). In both Denmark and Sweden, public schools were modelled to reduce class conflict and inequalities, not to bridge or bond ethno-religious differences.

In Sweden, it became popular to conceptualize and design citizenship education from a common value foundation in the 1990s. This approach marked a shift away from the post-War paradigm of scientific objectivism and Deweyan progressivism toward one which emphasized the contingency of education and knowledge on a greater context of values and norms (cf. Englund, 1999, p. 23f and 2000, p. 8f). This paradigmatic shift found its most explicit expression in the *Curriculum Committee* which delivered its final report in 1992 (SOU, 1992:94), resulting in the 1994 curriculum (Lpo 94, 1994). According to the committee, international migration, increasing diversity and the growing interdependence between societies called for a new philosophy of education that prepares citizens-to-be for a less predictable and rapidly changing world of international mobility and inter-cultural relations (SOU, 1992:94, p. 57ff). The committee emphasized liberal-democratic values and solidarity as the backbone of compulsory schooling. Still, its main source of inspiration was a von Humboldtian conception of *bildung*, stressing the generic abilities, personal growth and cultural continuities that schools’ fostering activities should aim for (SOU, 1992:94, p. 57).

The following decades are distinguished by a growing awareness of cultural diversity and its challenges. The first years of the 1990s bear the mark of the *Curriculum Committee’s* preoccupation with cultural continuity and can to considerable extent be attributed to the influence of a conservative-led center-right government (1991–1994) and a general sense of insecurity in the wake of European turmoil and economic recession. The following dozen years of social democratic reign (1994–2006), are characterized by a more pluralist approach in which the value foundation becomes the subject of deliberation, negotiation and change, rather than being derived from a set of relatively fixed reference points (Ekman, 2011, p. 40ff, Skolverket, 1998, 2000, Zachari & Modigh, 2003). This open-ended search for values is combined with a growing focus on ethnic discrimination and (structural) racism which continues after the regime change to a conservative-led center-right government (2006–14). Especially noteworthy is what may be called a universalization of the value foundation through the incorporation of human and children’s rights declarations, mandatory plans of anti-discrimination and equal treatment, all of which essentially serve to protect a liberal value of the individual (Fernández, 2012; cf. SOU, 2002:43; SOU, 2004:50; Prop. (Bill), 2005/06:38).

Turning to Denmark, a similar concern for increasing globalization was part of the reason for moving away in 1993 from the so far ‘cultureless’ purpose clause of the Danish comprehensive school law (*Folkeskoleloven*). It was changed to also state that schools ‘must make students intimate with Danish culture and contribute to their understanding of other cultures’ (L 509 1993).^4^ Bertel Haarder, Minister of Education until January 1993, later claimed ownership to this formulation (Hardis, 2002)—although no one objected to it in the parliamentary debate. He argued that it was meant to set the school apart from multiculturalism, which is consistent with the general public debate at the time where multicultural notions of citizenship was coming increasingly under fire.

Yet, discussion about citizenship education quickly gave way for other pressing issues. It was not until the late 1990s that then Minister of Education, Margrethe Vestager of the Social Liberal Party (*Radikale Venstre*), sought to revive local discussions on citizen-rearing through a commissioned report on democracy in the education system, two publications from the Ministry, and a law that gave students representatives in all general school councils and committees (Folketinget, 1999; Undervisningsministeriet [Ministry of Education], 1997, 1999, 2000). All documents address the importance of the national community in an increasingly globalized world. They also advance the notion of democracy as being a fragile culture or way of life that must continuously be reproduced in new generations and incorporated as a basic principle of everyday life. More interestingly, the documents also touched upon the negotiability of the national identity and how values should be open to contestation. This, however, did not gain traction in the scarce public debate that followed.^5^

The new center-right government that took office in 2001 had a more culturally conservative agenda for citizenship education. The notion of national identity they promoted was organic and static and critical discussions and reconstructions were de-emphasized. Throughout these years, the need for citizenship education became increasingly one-sidedly connected to Muslim students’ alleged lack of understanding and knowledge of democratic norms and Danish culture (Haas, 2008; Horst & Gitz-Johansen, 2010).

## Mother-tongue and bilingual instruction

Both Sweden and Denmark introduced mother-tongue instruction in 1976, granting pupils with immigrant background the right to education in their home language a few hours a week. In both cases, the reforms were motivated by both instrumental and intrinsic reasons; the fundamental value of the first language for learning a second language and for the development of the child’s self-identity, respectively. However, there are also notable differences between the two countries.

The Swedish mother-tongue instruction reform was animated by the recently introduced multicultural policy, according to which integration should rely on voluntary and mutual adaptation, and on the active recognition and endorsement of minority cultures (cf. SOU, 1974:69). The reform was widely supported across the party spectrum and portrayed as a win-win, for the parents, society and, most importantly, the children. Mother-tongue instruction was seen as a pre-emptive measure against ‘half-lingualism’ and alienation, since it helped children to develop language proficiency in and identification with both Swedish and the language of the parents (SOU, 1974:69, p. 241f; Borevi, 2002, p. 202–212). In Denmark, the political reasons were different. The primary reason for training children with immigrant background in their home language and culture was the perspective of repatriation (Mouritsen, Lex, Lindekilde, & Olsen, 2009, p. 75). At this point, immigrants were still mainly considered as guest-workers on a temporary stay. The secondary reason was that the ECC was preparing a directive giving children of member country citizens a right to receive teaching in their native language no matter where in the ECC they resided. The ECC recommended not discriminating between citizens and non-citizens of ECC member countries (ECC 1977).

In both countries, mother-tongue instruction was a rather unregulated, laissez-faire subject without syllabus. In the early 1990s a process of regularization commences, although with different outcomes. In Sweden, this process starts with the 1991 *Curriculum committee*, which connects bilingualism and inter-culturalism to the specific challenges and opportunities of internationalization. According to the committee, the pupils of mother-tongue instruction, together with their parents and teachers, ‘are a valuable and unique asset in the schools’ work with internationalization’ and for ‘creating understanding and solidarity with different peoples and cultures in the world’ (SOU, 1992:94, p. 214, cf. SOU, 1996:143, p. 158f). In the years that followed, the subject was consequently upgraded through a series of amendments. In 1994 it became a subject in its own right with a proper syllabus (Prop. (Bill), 1992/92:220). In the late 1990’s, the name mother-tongue replaced the previously used term home language to underscore the status of the subject, now placed on equal footing with Swedish, and the previous limit of seven years expanded to encompass the whole duration of the pupil’s school time (SOU, 1996:143, Prop. (Bill), 1997/98:94). In the latest school law of 2010, the school’s obligation to offer mother-tongue instruction was upgraded from regulation to law and simultaneously reinforced in pre- and secondary education (Prop. (Bill), 2009/10:165).^6^

In Denmark, the process of regulation took a different path. Denmark followed the ECC directive until 2002 where the centre-right government at the time abolished the right to mother-tongue instruction for non-EU citizens. Yet, as Kristjánsdóttir (2006) argues, up until 1993, when a center-left government took office for the first time in 11 years, mother-tongue instruction met strong resistance from within the Ministry of Education, which never started to develop pedagogical guidelines. Already in 1995, the Conservatives (*Det Konservative Folkeparti*) presented a parliamentary motion to remove the right to mother-tongue instruction (Folketinget, 1995). This gained support from the Liberal Party (*Venstre*) and a few years later the Social Democrats (*Socialdemokraterne*) as well adopted this position (Krohn, 1996). However, it did not become government policy because of resistance from the Social Liberal Minister of Education.

When guidelines were finally published in 2001, they both mentioned the instrumental value of mother-tongue instruction for the learning of Danish, *and* the intrinsic value it has for the self-identification and development of the child (Undervisningsministeriet [Ministry of Education], 2001). Yet, in the 2002 parliamentary debate only the representatives from the far-left Red-Green Alliance (*Enhedslisten*) and the Social Liberal Party (*Radikale Venstre*)—two minor parties—mentioned both of these values in their support for keeping the right (Folketinget, 2002). The Conservatives and the Liberal Party both argued, first, that no scientific evidence supported the instrumental value of mother-tongue instruction for learning Danish, and, secondly, that questions of cultural diversity and the pupil’s cultural identity belonged to the private sphere, not the school. Ulla Tørnæs, then Minister of Education, further explained that it’is in conflict with the value foundation of the Danish school to teach in another language than Danish [sic]’ (quoted in Folketinget, 2002). The Social Democrats did not argue in terms of culture and identity, but they questioned the evidence against mother tongue instruction improving Danish skills. However, unlike earlier, this uncertainty regarding its effect was now a reason to keep the right, they argued.

The debate is particularly illuminating in revealing ideological reasoning since the few Danish effect studies that have been conducted offer rather inconclusive evidence (Bjerg, 2002; Mehlbye, Rangvid, Larsen, Frederiksson, & Nielsen, 2011). Moreover, these studies only focus on the effect on learning, not on identity and self-esteem. Only the Red-Green Alliance and the Social Liberal Party argue, today as then, that it is a legitimate aim of the Danish school to pursue a multicultural agenda of strengthening and increasing awareness of the cultural duality of children of immigrants. This shows how discredited multicultural arguments are in Danish school politics. In fact, the right to mother-tongue instruction has not been pursued in any parliamentary motions or law proposals since 2002.

Contrary to Denmark, political parties of all colors in Sweden have viewed publically sanctioned classes in mother-tongue as vital to the intellectual and personal development of children with immigrant backgrounds *as well as* favorable to their integration into the majority society. And the scientific evidence has been viewed as strongly in favor (cf. Borevi, 2002: ch. 5; Hyltenstam & Tuomela, 1996). The main point of political controversy, rather, has been the extended application of bilingualism to foreign language teaching in the regular curriculum subjects. The Swedish school law allows schools to offer pupils with immigrant background between grades one to six and up to 50% of the total teaching time in a foreign language (Skolinspektionen, 2015). Traditionally, this is an opportunity that has been offered to children with Finnish background.^7^ Since it is an issue over which local authorities have full discretion, party-political struggles tend to erupt in local rather than national fora. A general pattern, however, is that regular curriculum classes in Arabic have been initiated by left-wing parties and opposed by the right, especially the Conservatives (*Moderaterna*). The proponents have typically invoked the same arguments as in the case of mother-tongue instruction, while the opponents point to the alleged detrimental effects to integration that separate teaching of native and immigrant children implies (see Wigerfelt, 2011).

## Religion

Public schooling in Denmark and Sweden has since its inception in the 19th century undergone a prolonged divorce between church and school, culminating after World War II. Their respective models of secular, non-confessional education differ, however. In Denmark, the subject Christianity Studies (*Kristendomskundskab*) is mandatory and taught at every level in primary school.^8^ It has been this way for at least the last 45 years. The evangelical-Lutheran faith of the Danish state church has always been at the center of the subject’s curriculum—as decided by the comprehensive school law—although the subject is non-confessional. Since 1975, the law stated that ‘the central knowledge area of Christianity Studies is the evangelical-Lutheran Christianity of the Danish state church’ (LBK 1534 2015). In 1993 it was added that ‘in the oldest classes the teaching must as well encompass foreign religions and other world views’ (L 509 1993). Since 1995, the Ministry of Education has defined the purpose of the subject in the following way: ‘The students must achieve knowledge of biblical stories and understanding of the importance of Christianity for the values in our cultural sphere’ (Undervisningsministeriet [Ministry of Education], 1995a, 2009a).

In Sweden, religion has been taught as a non-confessional subject in an objective and descriptive way since the late 1940s, and since the 1969 curriculum (Lgr 69, 1969) with a focus on world religions, as opposed to a prior focus on Christianity. Education in religion is largely comparative and inter-cultural in as much as it strives to enhance the pupil’s understanding of other cultures, belief systems, and ways of life. The stated objective is to ‘make pupils aware of how people in different religious traditions live with, and express, their religion and faith in different ways’ and to ‘comprehensively shed light on the role religions can play in society, both in peace efforts and conflicts, to further social cohesion and as a cause of segregation’ (Lgr 11, 2011, p. 186). Although much less pronounced than in Denmark, the syllabus also includes a particular focus on how ‘Christian traditions have affected Swedish society and its values’ (Lgr 11, 2011, p. 186).

Sweden’s secular and cosmopolitan approach to education in religion is combined with an ethical approach, which aims to ‘stimulate pupils to reflect on different life questions, their identity and their ethical attitudes’ (Lgr 11, 2011, p. 186). This focus on ethics can be traced back to the aforementioned *Curriculum Committee* (SOU, 1992:94) that gave a normative, value-centered and lasting impulse to the whole curriculum. In a controversial and much discussed passage, it stresses the importance of the ‘Christian tradition’ to the protection and reproduction of an ethics of ‘righteousness, generosity, tolerance and sense of responsibility’ (Lpo 94, 1994, p. 3; Lgr 11, 2011, p. 7). The passage was clearly an agonizing concession to the Christian Democrats (*Kristdemokraterna*), who enjoyed their first term in office ever, on behalf of the other three parties of the governing coalition. Today it serves as a peculiar reminder of that political compromise and of a preoccupation with values and ethics (Linné, 2001).

Immigration and the resulting increase in religious diversity create tensions in the selection of subject matters and the mode of teaching. Because of the clear exclusion of other religions, the Danish Christianity Studies has become the most contentious subject in the primary and lower secondary school. Still, it was not addressed as problematic by the Minister of Education, Margrethe Vestager (the Social Liberal Party), when she initiated the aforementioned values debate at the turn of the century. With the new center-right government in 2001, the importance of Christianity Studies, as well as History and Danish, became increasingly emphasized. Minister of Culture to be, Brian Mikkelsen (the Conservatives), defended Christianity Studies saying:

I am convinced that our democracy, our culture, our welfare, and our view of humanity, is closely tied to the national sentiment, which again is inextricably linked to Christianity. Even to Protestantism. Without an understanding of the Danish Christian history one can only shallowly acquire a Danish national identity (Mikkelsen, 2001).

In 2004, the Minister of Education, Ulla Tørnæs (Liberal Party), decided that a student’s exemption from Christianity Studies requires that the parents attend a meeting with the school’s principal in order to inform them of the subject’s content and the consequences of exemption. Among other things, the principal must inform them that Christianity Studies help students to become intimate with Danish culture (BEK 809 2004). Bertel Haarder (Liberal Party), who took office as Minister of Education in 2005, continued the defense and strengthening of Christianity Studies. He compared the subject’s cultural importance to that of H.C. Andersen and Karen Blixen (Klingsey, 2005) and claimed that Danes are ‘bound together by a common history which has led us to where we are now, and by a common culture which the church and Christianity has a big part in’ (Haarder, 2008). He continued by in 2006 adding Christianity Studies to the list of subjects students could be examined in when finishing 9th grade. Later in 2006, the government reformed the teacher education and added a new mandatory course called ‘Christianity Studies/Life Enlightment/Citizenship’ (*Kristendomskundskab/Livsoplysning/Medborgerskab*) with the purpose of securing that teachers know’the basic democratic values and Danish democracy, and are able to pass these values on to the school’ (Folketinget, 2006). The coupling of citizenship and Christianity Studies was not contested in the parliamentary debate by the left-wing parties. This is somewhat surprising, though, since the Red-Green Alliance, the Socialist People’s Party (*Socialistisk Folkeparti*), and the Social Liberal Party all argued from a secular standpoint, then and today, that Christianity Studies should change name to Religion or Ethics (*Livsanskuelsesundervisning*), and stop giving Christianity a privileged position. Although the Social Democrats agreed about changing the name, they did not want to significantly alter the content of the subject (Antorini, 2010). Still, nothing changed from 2011 to 2015, when the Social Democrats formed government with the Social Liberal Party and the Socialist People’s Party (the latter only until 2014).

In both Denmark and Sweden, charter schools with confessional orientation are controversial, although they are required to supply a public school equivalent education. Roughly 16% of all Danish students in primary and lower secondary education attend a charter school (Undervisningsministeriets databank 2015) compared to 14% of the Swedish students (Skolverket, 2015). Denmark has a long tradition of charter schools (literally termed ‘free schools’) going back to the mid-1800s, while it in Sweden is largely an innovation of the school reforms of the early 1990s. Through law changes in 2002, 2005, and 2006, Danish charter schools now experience increased monitoring and are required to provide civic education and inculcate a democratic ethos. These changes were largely driven by anxiety about fundamentalist ideas proliferating in Muslim charter schools (Olsen, 2015). In Sweden, calls for a more restrictive policy have been frequent, especially from the Liberal Party, and steps have been taken to enable stronger public control— most notably in the 2010 school law. Apart from allegations of increasing inequalities and segregation, a question of great concern is the effects of social and cultural stratification on the common social fabric of citizen formation. This applies especially to confessional schools, which tend to attract children from families with similar religious beliefs and are often believed to socialize children in ways that deviate significantly from the values and norms of public schools. While this is a serious concern for all the political parties, only the Sweden Democrats believe that such schools should be abolished altogether with the potential exemption of Christian schools. But Sweden’s ratification of the European Convention of Human Rights precludes a complete ban on confessional schools, which is also what the parties tend to say when pressed on the matter.^9^

## History and civics

Since the late 1970s, History has been systematically upgraded in the Danish school. From only being obligatory in the 8th and 9th grade, it is now obligatory from the 3rd till 9th grade, it has been added more weekly lessons,^10^ and been included on the list of subjects students can be examined in when finishing 9th grade. The civics subject has also been included on this list, but continues to be obligatory in only the 8th and 9th grade—which has not changed since 1979. This demonstrates the increased attention and importance that especially History has been given in the Danish school, chiefly driven by the goal of making students intimate with Danish culture. Bertel Haarder (the Liberal Party), Minister of Education 1982–1993 and 2005–2010, described History as one of the ‘culture carrying subjects’^11^ and argued that it ‘is about the people’s sense of self’ and must therefore ‘put special weight on the historical events that have been part of defining us as a people’ (Haarder quoted in Aagaard, 2005).

In Sweden, the relation between history and civics is quite the opposite. While the history subject gradually shrank over the latter half of the 20th century, civics^12^ became the citizen-rearing subject *par preference* as democratic literacy replaced patriotism in the curriculum (Zander, 1997; Hallenius, 2011, p. 59). Larsson (2001, p. 41) estimates a reduction of history teaching with more than 50% in 9th grade—and an even greater one in upper secondary (non-compulsory) education. Since 1989, there is no fixed rate for either subject but a lump of hours for all society-oriented subject (History, Civics, Religion, and Geography) that may not be reduced with more than 20% (SFS 1985:1100; SFS 2010:800). Parallel to this reduction of syllabus time, however, there have been recurring attempts to strengthen the history subject, especially from the right, and targeted campaigns to raise awareness and knowledge of particular historical events.

The objectives of History and Civics differ markedly in Denmark and Sweden, with the former representing a more conservative community-building/reproducing approach than the latter. The Danish ministerial curriculum plan of 2009 states that history is used ‘to build and strengthen the social cohesion in communities such as the national’ (Undervisningsministeriet [Ministry of Education], 2009b, p. 18). It further states that ‘historical narratives are crucial for the formation of identity’ and ‘offers obvious possibilities for empathy and identification’ (Ibid, p. 21). However, this emphasis on national cohesion also constitutes a strong move towards a very fixed, non-critical approach to the curriculum with greater emphasis on transmittance and maintenance of national culture instead of a reconstructive or transformative approach to national identity.

In Sweden, the objectives of history and civics are more comparative and cosmopolitan, but seemingly also more sensitive to normative considerations. The syllabus for History states that the education shall give pupils the ‘conditions to acquire an historical frame of reference and in-depth understanding of the present’, and that they shall develop a ‘chronological overview of how women and men have created and changed societies and cultures over time’ (Lgr 11, 2011, p. 172). In Civics, democracy and human rights are central themes that transcend most other topics, the aim being to enable pupils to acquire ‘knowledge of, and the ability to reflect on, values and principles that characterize a democratic society’ (Lgr 11, 2011, p. 199). It stresses both the generic ability to compare and contextualize, and the internalization of core substantial values such as a democratic spirit, tolerance, and gender equality.

Both countries have seen big campaigns to cultivate and enlighten the public through schools in the last 10 years. In Denmark, the History and Danish curriculum were partly standardized in 2009 by the incorporation of mandatory History and Danish canons. The aim was to strengthen historical consciousness and national identity.^13^ The Danish literature canon was presented already in late 2004 and included 14 Danish authors that students must read during their school time. In 2005, the committee to create a history canon started its work. The final list ignores the history of Danish immigration and emigration, reaffirms the importance of (some form of) cultural homogeneity, and ‘inspires the view that Danish history is characterized by a progression from peacefulness and justice to an even higher degree of peacefulness, liberality, justice, and modernism through non-violent steps and peaceful revolutions’ (Jørgensen 2014). Moreover, in 1995 the History curriculum plan stated that the task of the subject was to promote the student’s ‘insight into how humans are created by history as well as being creators of history’ (Undervisningsministeriet [Ministry of Education], 1995b). In the 2009 curriculum plan ‘creators of history’ was removed and instead the students should now know that they are created by history so they can ‘reflect on their opportunities of action’ (Undervisningsministeriet [Ministry of Education], 2009a), further downplaying a critical, reconstructive approach to national identity.

This idea of a canon was well received by the Social Democrats. Henrik Sass Larsen, a leading MP of the Social Democrats, voiced similar concerns in 2004 about the importance of historical knowledge for citizens to have a ‘common base’, and he also proposed a mandatory history canon (Jessen, 2004). He criticized progressive education for not caring about the national community and being elitist, arguing that ‘we need a common memory to remind each other about the values we share in Denmark’ (quoted in Hardis, 2004). In fact, the History canon mostly aroused public debate about the historical events selected, and later on only the Social Liberal Party argued against the whole idea of a canon saying it reflected a static, monocultural conception of the nation that simply was not true (Østergaard 2008). In fact, when a study in 2012 showed that many teachers fail to use the canons developed for History and Danish, the Minister of Education, Christine Antorini (the Social Democrats), was quick to stress that they must, downplaying any further need for discussion (Jessen, 2012). Although the left-wing parties now all acknowledge the usefulness of canons, they also agree that they should only be guiding, arguing that teacher autonomy is important (Folketinget, 2012). Still, the History canon remain mandatory today.

In Sweden, a similar attempt by a liberal MEP (Cecilia Wikström) to create a canon of Swedish prose and poetry was immediately shot down before making it past the editorials of the big newspapers. The very idea of a canonical Swedish literature was viewed as too hierarchical and excluding toward women and minorities (Fernández, 2011). Instead, the most noteworthy development was the massive information campaign by the Social Democratic government, spearheaded by then prime minster Göran Persson, on the atrocities of World War II and Nazism’s crimes against humanity. The campaign produced, among other things, a book about the Holocaust that was distributed to all pupils in secondary education—*Of this you may tell* (Bruchfeld & Levine, 1998)—.and a new government institution founded in 2003, *The Living History Forum* (Forum för levande historia), with the sole objective of spreading information and knowledge about the Holocaust, and to promote democracy, tolerance, and human rights to pupils and teachers. The campaign in general, and the forum in particular, were widely criticized for being ideologically biased and indoctrinating, since they focused exclusively on the crimes of right-wing but not left-wing totalitarian regimes. The forum’s assignment was consequently broadened to include both extremes of the ideological spectrum. Still, it continues to be questioned from both right and left, and above all by historians (cf. e.g. DN 2008).

All in all, history is an important source for citizenship education in both Denmark and Sweden, albeit in different ways. In Denmark, it has been used to highlight a selected set of defining moments, conditions, and developments to be proud of, while Swedish education in history seems to offer a repository of discouraging experiences, lessons and wisdoms to learn from. To put it simply, one is static and community-preserving, the other self-critical and self-examining.

## Concluding discussion

Let us return to the initial questions. What are defining ideas of citizenship education in the two countries? And, to what extent do they correspond with the general models of immigrant integration that prevail in citizenship and residence policies? The early 1990s brought new challenges to Denmark and Sweden—globalization, increasing international interdependence and, above all, unprecedented diversity in terms of the number of children with different immigrant backgrounds in schools—which had a strong and lasting impact on the development of school policy. The policy responses were quite different, however, and have served to exacerbate the divergence between the two countries. The Danish response has been to defend and fortify a monocultural approach to citizenship education, while the Swedish one has been further diversification and liberalization. In broad brush strokes, the defining idea of the former is to single out a national core culture to which minorities have to adapt in the process of integration, in which assimilation is viewed normatively and causally as a precondition for equal inclusion and opportunity. The defining idea of the latter, in contrast, is the recognition of multiple cultures that have to adapt to one another, rendering equal inclusion and opportunity contingent on the ability of each, especially the majority, to recognize the others.^14^ Although it is often believed and hoped that multiculturalism is a more effective model of integration than monoculturalism, this is essentially a different empirical question than the one we address here, which is the design and strength of public philosophies over time. So how consensual and stable are they?

The analysis shows that citizenship education policies in both countries have evolved under the influence of relatively consensual, stable and distinct public philosophies, albeit not without minor ‘cracks’ and deviations. The clearest example in the Danish case is mother tongue instruction, which does not really fit with the otherwise monocultural philosophy. It never enjoyed neither broad nor whole-hearted support, however, and becomes increasingly questioned in the 1990s until the early 2000s when the causal framework is no longer believed to support the normative, i.e. the positive correlation between bilingualism and integration. On this particular issue, then, the Danish philosophy changes from moderate to strong in the course of roughly a decade. On other issues we observe a gradual consolidation, if anything, of monoculturalism. The case of the literary and history canon is particularly interesting from this viewpoint as it epitomizes, arguably, both the strongest and most debated expression of monoculturalism. Clearly the idea in itself is strongly supported, although the selection of works can still provoke disagreement.

To the Swedish case pertains an equally consensual and stable philosophy. This philosophy is multicultural in its recognition of many cultures and mutual adaptation, although not to the point of preserving cultures and groups at the expense of integration, as we have seen in the debate on faith based charter schools. The normative framework is similar to the Danish, equal inclusion and opportunity, but largely detached from the existing nation-state with a focus on generic competences that citizens of the world, or maybe just of liberal democracies, require – tolerance and knowledge of the Other as this is conveyed in religion, history and civics. Even mother tongue instruction, which can be seen as a service to immigrant families rather than mainstream society, has become associated with the cosmopolitan virtues of modern Swedish citizenship. The equilibrium between pluralism and universalism is essential to the Swedish philosophy of integration, not just in schools but in general. If there are any signs of change, it is increasing universalism rather than accommodation of diversity.

The main aim of this article was not to develop Favell’s theory of public philosophies of integration, but to expand it beyond its usual habitat (a question that will be addressed in the next paragraph). For the purposes of the analysis, however, we have elaborated some elements in Favell’s theory to specify the conditions of strong, moderate and weak philosophies. Further development and testing of these conditions requires a large and more diverse sample than ours, and preferably a longer and/or more fluctuating time span. While both cases of our study demonstrate philosophical stability from the early 1990s to the present, which is what we expected, they also differ in interesting ways. The Danish political system is more politicized with greater ministerial autonomy and control over the administration, which seems to make philosophical stability highly contingent on *ideological convergence* between the main bloc parties. The Swedish political system is based on a sharp division between politics and administration with a much greater reliance on bureaucratic and academic expertise – of ‘what works’ – which seems to make philosophical stability more dependent on *pragmatic consensus*. The different outcomes with respect to mother tongue instruction illustrate this point well, in as much as reliance on empirical evidence, as opposed to normative convictions, seems to have been stronger in Sweden than Denmark.

Turning to the last question regarding the correspondence between the analysis of citizenship education politics presented here and other research on the Danish and Swedish politics of permanent residence and naturalization, our findings indicate a strong similarity. This is perhaps not surprising, but it suggests that scholars of (national) models of integration, and of the civic turn more specifically, should devote more attention to schooling and citizenship education. Sweden is a case in point, because it is often pictured as standing outside the West European turn towards emphasizing the civic integration of immigrants and their children. Yet our findings indicate that Swedish politics has also shown increased interest in how to form good, liberal-democratic citizens in response to international migration. While the phrase ‘civic integration’ is mainly associated with stricter conditions for permanent residence and citizenship, we believe that a broader and more variegated conception of the phenomenon is called for. Civic integration can be and is pursued by other means than formal tests and demands, and in other policy areas than immigration and naturalization.

It is plausible to assume, then, that a public philosophy of integration also configures which policy fields are associated with integration and what styles and methods. On this note, we argue that the alleged absence of a Swedish civic turn derive *both* from a leaner multicultural philosophy of adult integration *and* its relative separation from questions of entry and stay. In Sweden, the public debate on society’s ability to integrate immigrants have not dictated the design of entry, residence and naturalization requirements to the same extent as in Denmark and many other European countries. But it has influenced the form and content of citizenship education in schools. On this view, a civic assimilationist philosophy such as the Danish is more likely to transcend many policy areas, than a civic multiculturalist philosophy such as the Swedish, which largely relies on voluntary and mutual adaptation. In the latter case, civic integration policies which impose selected values and norms on citizens-to-be are easier to justify in public schools, which are compulsory and universal by definition, than in adult integration programs that single out migrants (but not natives) for such preparation. In a multicultural society, civic integration may be perfectly legitimate if it targets everyone, which of course is what public schools do. As long as the (official) end is to forge future able citizens of all children, immigrants, and natives, there is no contradiction between mandatory civic integration and multiculturalism. However, in a society like Denmark where assimilation is the accepted norm, it is legitimate to impose mandatory measures in whichever policy field seems relevant for the integration of immigrants. The act of singling out and subjecting minorities to such measures does not constitute any obvious form of discrimination, since it is understood in the first place that they are the ones who have to adapt to mainstream society.

## Footnotes

1 It should be noted, that we only introduce these concepts in order to describe the national approaches to integration. It is beyond this particular article to problematize or critically discuss them. Instead we aim to grasp the ideas that have shaped citizenship education politics in Denmark and Sweden.

2 If we did not assume ideational stability, we could produce 16 different scenarios if we treated the four dimensions (stability, consensus, normative and causal) as dichotomous. Most of these, however, would be scenarios in which one cannot speak of a public philosophy being significantly present.

3 Before reforms in the 1980s and 1990s, Sweden’s school system was considerably more centralized.

4 In 2006 it was moved to the fore of the clause.

5 Based on a broad search on infomedia.dk—a database of all Danish newspaper articles—in the month before and after the publication date.

6 The obligation only holds, however, if an adequate teacher is available and if there are at least five pupils who meet the requirements—at least one non-Swedish parent who speaks a foreign language at home, of which the pupil has at least some basic knowledge.

7 Finnish is one of Sweden’s five official minority languages, the other ones being Mienkiäli, Sami, Romani and Jiddish. Of these linguistic minorities the Finns are by far the largest.

8 Currently 2 lessons a week from 1st to 6th grade and 1 lesson a week in the remaining grades.

9 In a recent debate article, however, leading members of the Liberal party propose a complete ban on establishment of *new* confessional schools (Björklund et al., 2016).

10 The government sets a minimum for the number of lessons in History at each grade level. Today, it roughly corresponds to two lessons a week from fourth till eight grade and one lesson a week in third and ninth grade.

11 The others being Danish, Christianity Studies, and Civics.

12 The exact term of the civics subject in Sweden is social science (samhällskunskap) which includes both a ‘scientific’ and a ‘fostering’ approach to education.

13 The Ministry of Culture also published a culture canon in 2004. However, it was not incorporated into any curriculum plans.

14 The academic debate on whether and in what forms multiculturalism actually supports integration or segregation is ongoing (see e.g. Banting & Kymlicka, 2006; 2017). Fifteen years after the implementation of Swedish multiculturalism, Ålund and Schierup (1991) pointed out the signs of segregation and essentialization of minorities despite the inclusive and egalitarian intentions of the policy.

## References

[CR1] Aagaard, L. H. (2005). Overlæreren [The head teacher]. *Berlingske Tidende*, 20 Feb 2005.

[CR2] Ålund A, Schierup C-U (1991). Paradoxes of multiculturalism: Essays on Swedish society.

[CR3] Antorini, C. (2010). Danskhed i dag. Et forpligtende fællesskab [Danishness today. A binding community]. *Kristeligt Dagblad*, 21 Apr 2010.

[CR4] Banting K, Kymlicka W (2006). Multiculturalism and the welfare state. Recognition and redistribution in contemporary democracies.

[CR5] Banting K, Kymlicka W (2017). The strains of commitment. The political sources of solidarity in diverse socieities.

[CR6] BEK 809 (2004). Bekendtgørelse om procedureregler ved en elevs fritagelse for kristendomskundskab i folkeskolen [Act on rules of procedure regarding a student's exemption from Christianity Studies]. https://www.retsinformation.dk/Forms/R0710.aspx?id=24561. Accessed 17 Dec 2015.

[CR7] Bertossi C, Duyvendak JW (2012). National models of immigrant integration: The costs for comparative research. Comparative European Politics.

[CR8] Bjerg T (2002). Modersmålsforskere i et politisk minefelt [Mother-tongue instruction researchers in a political mine field].. Asterisk.

[CR9] Björklund, J., Avci, G., Malm, F., & Nylander, C. (2016). Vi vill sätta stop för flera religiösa friskolor i Sverige” [We want to put an end to more religious charter schools in Sweden]. *Dagens Nyheter*, 11 September 2016

[CR10] Borevi K (2002). Välfärdsstaten i det mångkulturella samhället [The welfare state in the multicultural society].

[CR11] Borevi K (2014). Multiculturalism and welfare state integration: Swedish model path dependency. Identities: Global Studies in Culture and Power.

[CR12] Bruchfeld S, Levine PA (1998). Om detta må ni berätta…: en bok om förintelsen i Europa 1933–1945 [This you must report…: a book on the holocaust in Europe 1933-1945].

[CR13] DN (2008). Regeringen gör historia till ideologiskt slagfält [The government makes history an ideological battle field]. *Dagens Nyheter*, 1 April 2008.

[CR14] ECC (1977). Council Directive of 25 July 1977 on the education of the children of migrant workers (77/486/ECC). http://eur-lex.europa.eu/legal-content/EN/TXT/PDF/?uri=CELEX:31977L0486&from=EN. Accessed 17 Dec 2015.

[CR15] Ekman J (2011). Skolan och medborgarskapet – en kunskapsöversikt [The school and citizenship – a survey of knowledge].

[CR16] Englund, T. (1999). Den svenska skolan och demokratin: möjligheter och begränsningar [The swedish school and democracy: possibilities and limitations]. In SOU, 1999:93 *Det unga folkstyret: Demokratiutredningens forskarvolym VI*. Stockholm: Kulturdepartementet.

[CR17] Englund T (2000). Deliberativa samtal om värdegrund – historiska perspektiv och aktuella förutsättningar [Deliberation on the value foundation – historical perspectives and current preconditions].

[CR18] Faas D, Hajisoteriou C, Angelides P (2014). Intercultural education in Europe: Policies, practices and trends. British Educational Research Journal.

[CR19] Favell A (2001). Philosophies of integration immigration and the idea of citizenship in France and Britain.

[CR20] Favell A, Guigni M, Passy F (2006). The nation-centered perspective. Dialogues on migration policy.

[CR21] Fernández C, Bevelander P, Fernández C, Hellström A (2011). Skolning till medborgarskap [Teaching citizenship]. Vägar till medborgarskap.

[CR22] Fernández C (2012). Liberaliseringen av svensk skolpolitik: en positionsbestämning [Positioning the liberalization of Swedish school policies]. Statsvetenskaplig tidskrift.

[CR23] Folketinget (1995). BSF 89 Forslag til folketingsbeslutning om indpasning af fremmedsprogede børn i det danske skolesystem [BSF 89 Proposal for a parliament resolution on the inclusion of foreign language children in the Danish school system]. https://www.retsinformation.dk/Forms/R0710.aspx?id=106931. Accessed 17 Dec 2015.

[CR24] Folketinget (1999). L 98 Forslag til lov om ændring af forskellige love på Undervisningsministeriets område. (Det repræsentative demokrati i uddannelsessystemet) [L 98 Law proposal to change different laws under the Ministry of Education (The representative democracy in the educational system]. http://webarkiv.ft.dk/?/Samling/19991/lovforslag_som_vedtaget/L98.htm. Accessed 17 Dec 2015.

[CR25] Folketinget (2002). L 142 Forslag til lov om ændring af lov om folkeskolen og lov om friskoler og private grundskoler m.v. (Modersmålsundervisning og sprogstimulering) [L 142 Law proposal to change the public school law and law on charter and private schools (Mother-tongue instruction and language stimulation)]. http://webarkiv.ft.dk/?/samling/20012/lovforslag_oversigtsformat/l142.htm. Accessed 17 Dec 2015.

[CR26] Folketinget (2006). L 220 Forslag til lov om uddannelsen til professionsbachelor som lærer i folkeskolen [L 220 Law proposal on the teacher education for public schools]. http://www.ft.dk/samling/20051/lovforslag/L220/index.htm#dok. Accessed 17 Dec 2015.

[CR27] Folketinget (2012). V 32 Om kanonlister i undervisningen [V 32 On canon lists in teaching]. http://www.ft.dk/samling/20111/vedtagelse/V32/index.htm. Accessed 17 Dec 2015.

[CR28] Goodman SW (2014). Immigration and membership politics in western Europe.

[CR29] Haarder, B. (2008). *Kirke, skole og kultur [Church, school, and culture]*. Speech at Sorgenfri kirke, 3 Apr 2008. www.uvm.dk. Accessed 4 Dec 2008

[CR30] Haas C (2008). Citizenship education in Denmark: Reinventing the nation and/or conducting multiculturalism(s)?. London Review of Education.

[CR31] Hallenius, M. (2011). *Clio räddar världen: En annalys av argumentationen för historieämnets ställning i det svenska skolsystemet i Historielärarnas Förenings Årsskrift, 1942–2004 [Clio Saves the World. An Analysis of the Argumentation for the Place of History in the Swedish school system in the Association of History Teachers Yearbook, 1942‐2004]*. PhD dissertation. Umeå: Umeå University.

[CR32] Hardis, A. (2002). Selvfølgelig er det diskrimination [Of course it is discrimination]. *Weekendavisen*, 1 Mar 2002.

[CR33] Hardis, A. (2004). Danmark for folket [Denmark for the people]. *Weekendavisen*, 6 Feb 2004.

[CR34] Hedetoft U, Vertovec S, Wessendorf S (2010). Denmark versus multiculturalism. The multiculturalism backlash: European discourses, policies and practices.

[CR35] Horst C, Gitz-Johansen T (2010). Education of ethnic minority children in Denmark: Monocultural hegemony and counter positions. Intercultural Education.

[CR36] Hyltenstam K, Tuomela V, Hyltenstam K (1996). Hemspråksundervisningen [Mother-tongue instruction]. Tvåspråkighet med förhinder? Invandrar- och minoritetsundervisningen i Sverige.

[CR37] Jensen, K. K. (2016). *Scandinavian Immigrant Integration Politics: Varieties of the Civic Turn*. PhD dissertation, Aarhus University.

[CR38] Jensen KK (2014). What can and cannot be willed: How politicians talk about national identity and immigrants. Nations and Nationalism.

[CR39] Jessen, B. (2004). Min generation er historieløs [My generation is without history]. *Berlingske Tidende*, 5 Apr 2004

[CR40] Jessen, B. (2012). Antorini: Lærere skal undervise i kanoner [Antorini: Teachers must teach the canons]. *Berlingske Tidende*, 12 Jan 2012.

[CR41] Joppke C (2007). Transformation of immigrant integration: Civic integration and antidiscrimination in the Netherlands, France, and Germany. World Politics.

[CR42] Jørgensen SL (2014). The history we need: Strategies of citizen formation in the Danish history curriculum. Scandinavian Journal of Educational Research.

[CR43] Klingsey, M. (2005). Folkeskole til kamp for åndsfrihed [Public school to fight for freedom of thought]. *Information*, 10 Apr 2005.

[CR44] Kristjánsdóttir, B (2006). Viljen til undervisning i tosprogede elevers modersmål [The will to teach multilingual student's home language]. In C. Horst (ed.), Interkulturel pædagogik. Flere sprog – problem eller ressource? (2nd ed.). København: Dafolo/Kroghs Forlag.

[CR45] Krohn, O. (1996). S: Indvandrere skal ikke lære eget sprog [S: Immigrants should not learn their own language]. *Aktuelt*, 19 Jul 1996

[CR46] L 509 (1993). Lov om folkeskolen [The public school law]. https://www.retsinformation.dk/Forms/R0710.aspx?id=74941. Accessed 17 Dec 2015.

[CR47] Larsson, H. A. (2001). Barnet kastades ut med badvattnet: Historien om hur skolans historieundervisning närmast blev historia [The child is thrown out with the bathwater: The story of how the school's teaching of history almost became history]. *Aktuellt om historia* 2001(02). Stockholm: Historielärarnas förening.

[CR48] LBK 1534 (2015). Bekendtgørelsen af lov om folkeskolen [Act on the public school law]. https://www.retsinformation.dk/forms/r0710.aspx?id=176327. Accessed 17 Dec 2015.

[CR49] *Lgr 11* (2011). Läroplan för grundskolan, förskoleklassen och fritidshemmet 2011 [Curriculum for the primary school 2011]. Stockholm: Skolverket.

[CR50] *Lgr 69* (1969). *Läroplan för grundskolan 1969 [Curriculum for the primary school 1969]*. Stockholm: Skolöverstyrelsen.

[CR51] Linné A, Linde G (2001). Moralfostran i svensk obligatorisk skola [Moral education in Swedish compulsory schooling].. Värdegrund och svensk etnicitet.

[CR52] *Lpo 94* (1994). 1994 års läroplan för det obligatoriska skolväsendet [Curriculum for the primary school 1994]. Stockholm: Skolverket.

[CR53] Mehlbye J, Rangvid BS, Larsen BØ, Frederiksson A, Nielsen KS (2011). Tosprogede Elevers Undervisning I Danmark Og Sverige [The teaching of bilingual students in Denmark and Sweden].

[CR54] Mikkelsen, B. (2001). Det kontroversielle skolefag [The controversial school subject]. *Politiken*, 25 Aug 2001.

[CR55] Mouritsen P, Modood T, Triandafyllidou A, Zapata-Barrero R (2006). The particular universalism of a Nordic civic nation: Common values, state religion and Islam in Danish political culture. Multiculturalism, Muslims and citizenship: A European approach.

[CR56] Mouritsen P, Mouritsen P, Jørgensen KE (2008). Political responses to cultural conflict: Reflections on the ambiguities of the civic turn. Constituting communities: Political solutions to cultural conflict.

[CR57] Mouritsen P, Olsen TV (2013). Denmark between liberalism and nationalism. Ethnic and Racial Studies.

[CR58] Mouritsen, P., Lex, S., Lindekilde, L., & Olsen, T. V. (2009). Immigration, integration and the politics of cultural diversity in Denmark: Political discourse and legal, political and educational challenges. Integrated country report for the project *EMILIE - A European approach to multicultural citizenship: Legal, political and educational challenges*.

[CR59] Olsen TV (2015). The Danish free school tradition under pressure. Comparative Education.

[CR60] Østergaard, M. (2008). Hvorfor skal vi være dansk-på-dåse? [Why must we be Danish-in-a-can?] *Politiken,* 26 Jun 2008

[CR61] Prop. (Bill) 1992/92:220, *En ny läroplan för grundskolan och ett nytt betygssystem för grundskolan, sameskolan, specialskolan och den obligatoriska särskolan*. [A new curriculum for the primary school etc.]

[CR62] Prop. (Bill) 1997/98:94, *Läroplan för det obligatoriska skolväsendet, förskoleklassen och fritidshemmet m.m. [Curriculum for compulsory schooling etc.]*

[CR63] Prop. (Bill) 2005/06:38, *Trygghet, respekt och ansvar – om förbud mot diskriminering och annan kränkande behandling av barn och elever. [Safety, respect and responsibility - about prohibiting diskrimination and other offensive treatment of children and students]*

[CR64] Prop. (Bill) 2009/10:165, *Den nya skollagen – för kunskap, valfrihet och trygghet*. [The new school law - for knowledge, freedom of choice and safety]

[CR65] SFS (Swedish law) 1985:1100, *Skollag [School law]*

[CR66] SFS (Swedish law) 2010:800, *Skollag [School law]*.

[CR67] Skolinspektionen (2015). Tvåspråkig undervisning [Bilingual teaching]. http://www.skolinspektionen.se/sv/Tillstandsprovning/Tvasprakig-undervisning-pa-elevens-umgangessprak/. Accessed 18 Dec 2015.

[CR68] Skolverket (1998). *Skolan och värdegrunden Skolornas arbete med värdefrågor – mål, verksamhet och utvärdering i offentliga och fristående skolor [The school and values education. The school’s work with value questions – goals, activities and independent schools]*.

[CR69] Skolverket. (2000). *En fördjupad studie om värdegrunden. Om möten, relationer och samtal som förutsättningar för arbetet med de grundläggande värdena [An in-depth study of values education. About meetings, relations and conversations as the precondtions for working with the basic values]*. Stockholm: Skolverket.

[CR70] Skolverket (2015) *Tabell 2a: Skolenheter och elever läsåren 2009/10 till 2014/15. [Table 2a: School units and students in the years 2009/10 to 2014/15]*. http://www.skolverket.se/statistik-och-utvardering/statistik-i-tabeller/grundskola/skolor-och-elever. Accessed 18 Dec 2015.

[CR71] Soininen M (1999). The ‘Swedish model’ as an institutional framework for immigrant membership rights. Journal of Ethnic and Migration Studies.

[CR72] SOU 1974:69. *Invandrarutredningen 3. Invandrarna och minoriteterna* (huvudbetänkande av invandrarutredningen) [Immigrant report 3. Immigrants and minorities [main report from the immigrant committee]. Stockholm: Arbetsmarknadsdepartementet.

[CR73] SOU 1992:94. *Skola för bildning* (betänkande av läroplanskommittén) [School for Bildung (report from the curriculum committee)]. Stockholm: Utbildningsdepartementet.

[CR74] SOU 1996:143. *Krock eller möte: Om den mångkulturella skolan* (delbetänkande av skolkommittén) [Clash or meeting: About the multicultural school (interim report from the school committee)]. Stockholm: Utbildningsdepartementet.

[CR75] SOU 2002:43. *Ett utvidgat skydd mot diskriminering [An increased protection from discrimination]*. Stockholm: Justitiedepartementet.

[CR76] SOU 2004:50. *Skolans ansvar för kränkningar av elever [The schools responsibility for students being violated]*. Stockholm: Utbildningsdepartementet.

[CR77] Telhaug AO, Mediås OA, Aasen P (2004). From collectivism to individualism? Education as nation building in a Scandinavian perspective. Scandinavian Journal of Educational Research.

[CR78] Telhaug AO, Mediås OA, Aasen P (2006). The Nordic model in education: Education as part of the political system in the last 50 years. Scandinavian Journal of Educational Research.

[CR79] Undervisningsministeriet [Ministry of Education] (1995). Kristendomskundskab Faghæfte 3 [Christianity Studies Subject Book 3].

[CR80] Undervisningsministeriet [Ministry of Education] (1995). Historie Faghæfte 4 [History Subject Book 4].

[CR81] Undervisningsministeriet [Ministry of Education] (1997). Det repræsentative demokrati i uddannelsessystemet [The representative democracy in the educational system].

[CR82] Undervisningsministeriet [Ministry of Education] (1999). Uddannelse og Fællesskab [Education and Community].

[CR83] Undervisningsministeriet [Ministry of Education] (2000). Værdier i virkeligheden [Values in reality].

[CR84] Undervisningsministeriet [Ministry of Education] (2001). Modersmål for tosprogede elever: Faghæfte 34 [Mother-tongue for bilingual students: Subject book 34].

[CR85] Undervisningsministeriet [Ministry of Education] (2009). Fælles Mål - Kristendomskundskab - Faghæfte 3 [Common Goals - Christiany Studies - Subject Book 3].

[CR86] Undervisningsministeriet [Ministry of Education] (2009). Fælles Mål – Historie – Faghæfte 4 [Common Goals - History - Subject Book 4].

[CR87] Undervisningsministeriets databank (2015). http://www.uvm.dk/databanken. Accessed 17 Dec 2015.

[CR88] Wiesbrock A (2011). The integration of immigrants in Sweden: A model for the European union?. International Migration.

[CR89] Wigerfelt B (2011). Undervisning på svenska och arabiska tar form i ett mångetniskt område [Teaching in Swedish and Arabic takes shape in a multi-ethnic area]. Educare.

[CR90] Zachari G, Modigh F (2003). Värdegrundsboken: om samtal för demokrati i skolan [The value education book: on deliberation before democracy in school].

[CR91] Zander U (1997). Från nationell till demokratisk ideologi eller berättelsen om samhällskunskapens uppgång och historieämnets fall [From national to democratic ideology or the story of the rise of civic education and the fall of the history subject]. Utbildning och demokrati.

